# Berberine Ameliorates High Glucose-Induced Cardiomyocyte Injury via AMPK Signaling Activation to Stimulate Mitochondrial Biogenesis and Restore Autophagic Flux

**DOI:** 10.3389/fphar.2018.01121

**Published:** 2018-10-03

**Authors:** Weijian Hang, Benhong He, Jiehui Chen, Liangtao Xia, Bing Wen, Tao Liang, Xu Wang, Qianying Zhang, Yue Wu, Qingjie Chen, Juan Chen

**Affiliations:** ^1^Department of Biochemistry and Molecular Biology, School of Basic Medicine and the Collaborative Innovation Center for Brain Science, Tongji Medical College, Huazhong University of Science and Technology, Wuhan, China; ^2^Institute for Brain Research, Huazhong University of Science and Technology, Wuhan, China; ^3^Key Laboratory of Neurological Disease of National Education Ministry, Tongji Medical College, Huazhong University of Science and Technology, Wuhan, China; ^4^Department of Cardiovascular Medicine, Lichuan People’s Hospital, Lichuan, China; ^5^Hubei Key Laboratory of Genetics and Molecular Mechanisms of Cardiological Disorders, Huazhong University of Science and Technology, Wuhan, China; ^6^New Products of TCM Senile Diseases Co-Innovation Center of Hubei, School of Basic Medicine, Hubei University of Chinese Medicine, Wuhan, China

**Keywords:** berberine, cardiomyocyte hypertrophy, mitochondrial, high glucose, fragmentation, diabetes mellitus

## Abstract

**Background:** Type II diabetes (T2D)-induced cardiomyocyte hypertrophy is closely linked to the impairment of mitochondrial function. Berberine has been shown to be a promising effect for hypoglycemia in T2D models. High glucose-induced cardiomyocyte hypertrophy *in vitro* has been reported. The present study investigated the protective effect and the underlying mechanism of berberine on high glucose-induced H9C2 cell line.

**Methods:** High glucose-induced H9C2 cell line was used to mimic the hyperglycemia resulting in cardiomyocyte hypertrophy. Berberine was used to rescue in this model and explore the mechanism in it. Confocal microscopy, immunofluorescence, RT-PCR, and western blot analysis were performed to evaluate the protective effects of berberine in high glucose-induced H9C2 cell line.

**Results:** Berberine dramatically alleviated hypertrophy of H9C2 cell line and significantly ameliorated mitochondrial function by rectifying the imbalance of fusion and fission in mitochondrial dynamics. Furthermore, berberine further promoted mitogenesis and cleared the damaged mitochondria via mitophagy. In addition, berberine also restored autophagic flux in high glucose-induced cardiomyocyte injury via AMPK signaling pathway activation.

**Conclusion:** Berberine ameliorates high glucose-induced cardiomyocyte injury via AMPK signaling pathway activation to stimulate mitochondrial biogenesis and restore autophagicflux in H9C2 cell line.

## Introduction

Diabetes has the distinguished manifestation of high blood glucose level, or the so-called hyperglycemia. Hyperglycemia is detrimental to blood vessels, kidney, heart, eyes, nerves, and many other organs (King, 2008; Lindholm et al., 2013). It was reported that hyperglycemia is a distinguished risk factor of cardiovascular events, and many diabetes patients have got cardiomyocyte hypertrophy, decreased heart function (Kuwabara et al., 2015), and eventually heart failure (Dei Cas et al., 2015). Although hyperglycemia is obviously related to myocardium hypertrophy, the precise mechanism behind it is still under investigation.

The emergence of hyperglycemia is the result of loss of control of energy metabolism. Mitochondria are the key organelle in cell responsible for controlling metabolism process (Porporato et al., 2016). Its normal function is vital to cell, especially to those are sensitive to energy condition like myocardium and neuron. Mitochondrial function is precisely regulated, just like mitochondrial fission and fusion process (Michalska et al., 2016). It is reported wildly that mitochondria are under ceaseless fission and fusion whole through the cell life and its morphology can somehow reflect mitochondrial function (Chan, 2012). The fission and fusion process is modulated by several important proteins. Mitofusion 1 (Mfn1) and mitofusion 2 (Mfn2) are responsible for the fusion of outer mitochondrial membrane (OMM), while optical atrophy 1 (OPA1) for inner mitochondrial membrane (IMM) (Farmer et al., 2018). As to mitochondrial fission, dynamic-related protein 1 (Drp1) is the key motor protein that governs mitochondrial fission (Otera et al., 2013). In addition, unlike those mitochondrial fusion proteins, Drp1 is mainly located at cytoplasm, and it needs several mitochondrial adaptors like mitochondrial fission factor (MFF) to be recruited to mitochondria (Zheng et al., 2018). It was reported that in hypertrophied myocardium, mitochondria showed over-fission morphology (Pennanen et al., 2014; Tang et al., 2014), which indicated that mitochondrial fission/fusion process may have relationship with myocardium hypertrophy, but the mechanism is still not very clear. What’s more, several articles have reported that hyperglycemia may induce myocardium hypertrophy via interfering mitochondrial normal function (Adeghate and Singh, 2014; Mali et al., 2016; Li et al., 2017). Hence, mitochondria may play an important role in progression of diabetic myocardium hypertrophy.

Hyperglycemia is the manifestation of metabolism imbalance. AMPK is the key modulator that regulates the state of cell energy supplement. AMPK can sense the change of ratio of ADP/ATP (Gorini and De Angelis, 2018) and regulate the activity of cell autophagy by regulating mTOR (Majd et al., 2018). The disturbance of AMPK by hyperglycemia can damage the function of mitochondria, and have direct effects on hypertrophy (Ren et al., 2010; Karbasforooshan and Karimi, 2017). Hence, targeting at AMPK is a promising way to treat hypertrophy. Berberine is the purified extracts of traditional Chinese medicine *Coptis chinensis*. It was reported that berberine can activate AMPK and have vast effects to several diseases including metabolic syndrome, cardiovascular diseases (Cicero and Tartagni, 2012), and neurodegeneration diseases (Xu et al., 2017). Several clinical trials investigating effects of berberine doses to metabolic syndrome and cardiovascular diseases have been registered on Clinical Trails, and those completed revealed good protecting effects of berberine (Gonnelli et al., 2015; Zhang et al., 2016; Ming et al., 2018). Although it was reported that berberine can attenuate hypertrophy induced by pressure overload, high fatty acid, and high insulin plus high glucose (Chang et al., 2013; Li M.H. et al., 2014), the exact effect of high glucose level does to the progression of hypertrophy is still not discussed. Here we provide results that berberine can attenuate hypertrophy of H9C2 cell line, a typical embryonic rat heart tissue cell line, by activating AMPK and help upregulating the level of autophagy, so recovering mitochondrial function by promoting mitogenesis and clearing the damaged mitochondria via mitophagy.

## Materials and Methods

### Materials

Following monoclonal antibodies were purchased from CST: Mfn1 (#14739), Mfn2 (#9482), OPA1 (#67589), Drp1 (#8570), p-Drp1 Ser 616 (#4494), MFF (#84580), Beclin1 (#3495), Atg5 (#12994), p62 (#23214), LC3A/B (#12741), mTOR (#2983), Parkin (#4211), AMPKα (#5831), and p-AMPK Thr172α (#2535). Following monoclonal antibodies were purchased from Abcam: β-actin (ab8826), PINK1 (ab75487), and p-mTOR Ser 2481 (ab137133). Following polyclonal antibodies were purchased from ABclonal: VDAC (A15735) and PGC1α (A12348). All of these above antibodies were used at 1:1000 dilution, besides β-actin (ab8826) at 1:5000 and PINK1 (ab75487) at 1:200. PVDF membrane was purchased from GE Health (10600023). D-Glucose (G7528) and berberine (PHR1502) were purchased from Sigma, Mdivi-1(ab144589) and compound C (ab120843) were purchased from Abcam.

### Cell Culture and Treatment

H9C2 cell line was purchased from ATCC and cultured with DMEM-low glucose (HyClone) in 37°C, 5% CO_2_. Medium was refreshed every day. When the cell confluency reached 80–90%, cells were passed by using trypsin-EDTA. For six-well cell culture plate, every 5 × 10^5^ H9C2 were passed into one well, and cultured overnight for attachment. Berberine were added into medium at the final concentration of 100 nM for 30 min pretreatment, then D-glucose was added at the final concentration of 50 mM for 24 h. H9C2 cells were pretreated with 5 nM Mdivi-1 for 30 min and underwent high glucose for 24 h. For compound C treatment, 1.25 μM of compound C was added together with 50 mM D-glucose after 30 min of berberine pre-treatment.

### Protein Extraction

About 10^6^ cells were washed by ice-old PBS with 1 mM PMSF added twice, and lysis by 100 μl RIPA (Tris–HCl, pH 7.5, 50 mM; NaCl, 150 mM; EDTA, 1 mM; 1% NP-40; 0.1% SDS; 1% Triton X-100) with protease inhibitor PMSF and phosphatase inhibitor cocktail added. Then the cell lysates were incubated on ice for 15 min with vortex for 30 s every 5 min. After incubation, 12 min of 12,000 × *g* centrifuge at 4°C was taken, and the supernatant was moved into a new Eppendorf tube and quantified the protein concentration with BCA Assay Kit (Thermo, #23225).

### Western Blotting

Western blotting was performed as described before (Xue et al., 2017). Each lane was loaded 20–40 μg protein, and the PVDF membrane was blocked with 5% skim milk in 0.1% TBST for 1 h. After washing the membrane for three times, the membrane was incubated with primary antibody at 4°C overnight. HRP-conjugated secondary antibody and ECL kit were used to detect primary antibody with Bio-Rad Exposure System. The gray value of each lane was calculated with ImageJ.

### RNA Isolation and RT-PCR

Total RNA were isolated with Trizol (Invitrogen) according to manufacturer’s protocol. The purification and concentration of RNA were measured with NanoDrop (Thermo), and 1 μg of total RNA was used to reverse transcription using reverse transcription kit (TOYOBO) according to manufacturer’s guidance. The resulting cDNA was 10 times diluted with DNase-free water followed by quantification of real-time PCR using Bio-Rad CFX96. All data were expressed as the relative ratio of target gene to the housekeeping gene GAPDH. The following primers were used for real-time PCR:

Actin – F: ATGACCCAAGCCGAGAAGG,
R: CGGCCAAGTCTTAGAGTTGTTG;
ANP – F: GCTTCCAGGCCATATTGGAG,
R: GGGGGCATGACCTCATCTT;
BNP – F: ATCTCCTGAAGGTGCTGTCC,
R: TCCAGCAGCTGCATCTTGAA;
GATA – F: GGGCCCTCTTTGTCATTCTTC,
R: TCCTTGCTTTCTGCCTGCTAC;
RCAN1.4 – F: CCCGTGAAAAAGCAGAATGC,
R: TCCTTGTCATATGTTCTGAAGAGGG;
NRF1 – F: TTTATGGCAGATCGTGCAGGT,
R: CGCTGTCTGATATCCTGGTGG;
NRF2 – F: CAACTACTCCCAGGTTGCCC,
R: AGTGACTGAAACGTAGCCGAA;
Tfam – F: TGATTCACCGCAGGAAAAGC,
R: CGAGTTTCGTCCTCTTTAGCA;
TFB1m – F: CGGAAAACTCAGCACTTGCC,
R: AGCCTCAAGTCCAGGAGGAA;
TFB2m – F: GTGGTTGCGCTCGAAAGTG,
R: CTCGAGAAGACATAGCAGGTGG;
COX1 – F: ATACCAAACGCCCCTCTTCG,
R: TGTTGAGGTTGCGGTCTGTT;
  28SrRNA – F: AGGACCCGAAAGATGGTGAACTA,
R: CGGAGGGAACCAGCTACTAGAT.

### Immunofluorescence

Cells were fixed with 4% paraformaldehyde for 5 min at room temperature; 0.1% Triton X-100 was used for 10 min to permeate cell membrane; and 5% BSA was added to the cell to block non-specific antigen recognition. After blocking for 30 min, primary antibody was used to detect intrinsic with a dilution of 1:200. Cells were plated at room temperature (20°C) for 4 h. For cytoskeleton staining, FITC-labeled phalloidin (Beyotime, #C1033) was used with a dilution of 1:200 at room temperature for 30 min. Then anti-rabbit IgG-Alexa Fluro 594 conjugate (CST, 8889), FITC-labeled goat anti-rabbit secondary antibody (ServiceBio, #GB22303), or Cy3-labeled goat anti-mouse secondary antibody (ServiceBio, #GB21301) was used to detect the primary antibody with a dilution of 1:200 for 1 h at 37°C. DAPI (Beyotime, #C1005) was used at last to stain nuclei. After that, cells were observed with Nikon Confocal microscopy.

### Measuring of Mitochondrial Membrane Potential (MMP)

Mitochondrial membrane potential was measured according to the manufacturer’s protocol (Beyotime, #C2006). In brief, after treatment, the medium was removed, and cells were washed with warmed-up PBS twice. Then cells were incubated in 1 ml 1× JC-1 staining working solution and 1 ml growth medium at 37°C for 20 min. After incubation, the cells were washed with 1× staining buffer twice and were observed with Nikon Confocal microscopy. For statistical analysis, each group was photographed randomly 10 fields and about 100 cells red and green fluorescence intensity were measured by ImageJ.

### Measuring of ATP

ATP was measured according to the manufacturer’s protocol (Beyotime, # S0026B). Briefly, cells were incubated with lysis buffer on ice and centrifuged at 12,000 × *g* for 5 min. The supernatant was used to measure ATP level according to standard curve using luminometer (Promega).

### Measuring of ROS Level

ROS level was measured according to the manufacturer’s protocol (Beyotime, #S0033). To be brief, DCFH-DA probe was diluted to 10 μM with FBS-free DMEM. Then cells were incubated with DCFH-DA probe at 37°C for 30 min. Then cells were observed with Nikon Confocal microscopy. For statistical analysis, each group was photographed randomly 10 fields and about 100 cells green fluorescence intensity were measured by ImageJ.

### Statistical Analysis

Data were analyzed by Graph Pad Prism 5 software and expressed as mean ± SEM. Statistical significance was assessed by one-way ANOVA following Tukey’s *post hoc* Test. *P-*values < 0.05 suggested that the level of significances was existed.

## Results

### Berberine Can Attenuate High Glucose-Induced Hypertrophy

**FIGURE 1:**
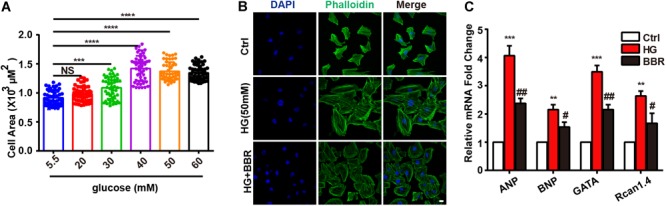
Berberine ameliorated high glucose-induced myocardium hypertrophy. **A**: Cell area of H9C2 after different 24 h glucose concentration treatment, about 100 cells for each group were measured. **B**: Representative confocal images of H9C2 cells stained with FITC-labeled phalloidin to show its area. Scale bar is 10 μm. **C**: Relative mRNA level of myocardium hypertrophy biomarkers quantified by real-time PCR. *n* = 3 for each group, each sample were measured in three replications. ^∗∗^*P* < 0.01 vs. Ctrl; ^∗∗∗^*P* < 0.001 vs. Ctrl; ^∗∗∗∗^*P* < 0.0001 vs. Ctrl; **^#^***P* < 0.05 vs. HG; **^##^***P* < 0.01 vs. HG.

Firstly, we found that high glucose treatment induced hypertrophy in H9C2 cells. As is shown in **Figure 1A**, H9C2 cell area became larger in response to high glucose treatment gradually, and reached to the largest at the concentration of 50 mM glucose. Then we used FITC-labeled phalloidin to stain actin cytoskeleton (**Figure 1B**), and found that H9C2 was larger under 50 mM high glucose treatment. In order to confirm the optimal concentration of berberine, we conducted CCK8 cell viability test. Results showed that berberine didn’t have significant effect on H9C2 cell viability unless the concentration is higher than 400 nM (**Supplementary Figure S1B**); this showed that berberine is safe under 400 nM. In order to minimize the toxic effects of berberine, we tended to find the minimum concentration that can activate AMPK, as was reported that berberine is an agonist of AMPK (Yerra et al., 2018). It was showed that 50 nM berberine can only slightly activate, but 100 nM berberine is sufficient to activate AMPK (**Supplementary Figures S1A,C,D**). We also measured the level of LDH in the medium of H9C2 cells, finding that LDH was upregulated after HG treatment, and 100 nM berberine was able to attenuate the upregulation of LDH, so 100 nM berberine was chosen to establish the cell model. Then we found that 100 nM berberine can attenuate H9C2 hypertrophy induced by high glucose treatment. Besides, berberine can downregulate mRNA expression of biomarkers of hypertrophy (**Figure 1C**), which indicated that it can indeed alleviate hypertrophy.

### Berberine Protected Mitochondria From Impairments of High Glucose Treatment

**FIGURE 2:**
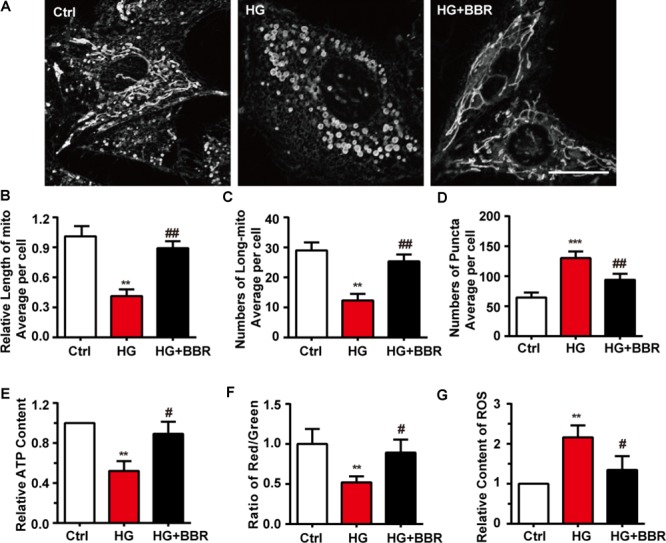
Berberine attenuated high glucose-induced mitochondria impairment by balancing fission and fusion. **A**: Representative confocal images of mitochondria of H9C2 cells stained with mitotracker. Scale bar is 10 μm. **B**: Statistical analysis of mitochondrial length using ImageJ, for each group, *n* = 40–50 cells were analyzed. **C**: Statistical analysis of mitochondrial area using ImageJ, for each group, *n* = 40–50 cells were analyzed. **D**: Statistical analysis of mitochondrial puncta using ImageJ, for each group, *n* = 40–50 cells were analyzed. **E**: Relative ATP production was measured. **F**: Relative MMP was measured by JC-1 and was quantified using the ratio of red and green fluorescence intensity. **G**: Relative ROS level was measured using DCFH-DA and were quantified using green fluorescence intensity, for each group, >10 fields were measured. ^∗∗^*P* < 0.01 vs. Ctrl; ^∗∗∗^*P* < 0.001 vs. Ctrl; **^#^***P* < 0.05 vs. HG; **^##^***P* < 0.01 vs. HG.

Next we used mitotracker, a live-cell-permeable mitochondria dye, and found that compared to control, mitochondria showed more broken-like shape under high glucose treatment (**Figure 2A**), and became smaller and shorter (**Figures 2B,C**). As a result, there were more mitochondrial puncta (**Figure 2D**) in high glucose treatment. In consistent with morphology changes, berberine can protect mitochondria from fragmentation (**Figures 2A–D**). Since mitochondrial morphology showed distinguished changes under the treatment of high glucose and berberine, we were interesting whether its functions were also impaired by high glucose. Like our speculation, ATP production (**Figure 2E**), MMP (**Figure 2F**) decreased, while ROS production (**Figure 2G**) increased after high glucose treatment, which were all signs of mitochondrial dysfunction. Still, berberine can reverse these detrimental changes and preserve mitochondrial function (**Figures 2E–G**).

### Berberine Attenuates High Glucose-Induced Mitochondrial Impairment by Balancing Fission and Fusion

**FIGURE 3:**
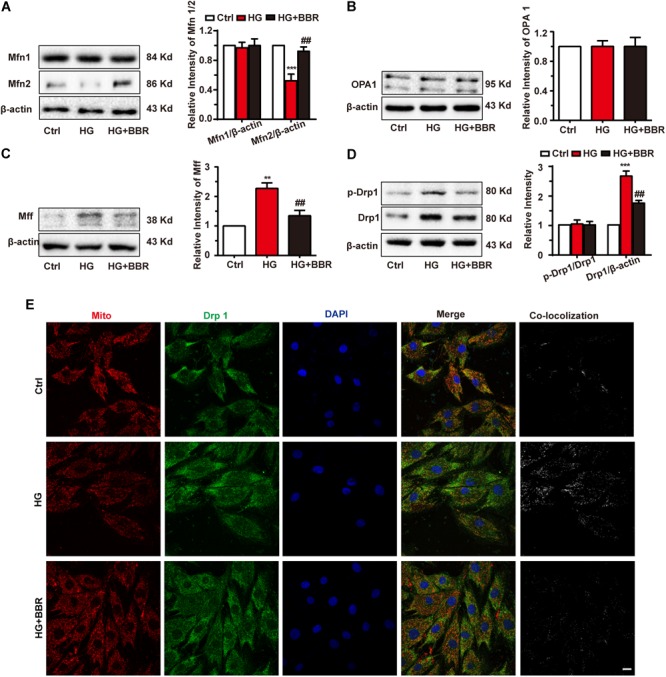
Berberine balanced mitochondrial fission and fusion in high glucose-induced H9C2 cells. Representative western blot results and quantification of Mfn1 and Mfn2 **(A)**, OPA1 **(B)**, and MFF **(C)**. Each western blot was conducted for three times. **D**: Representative western blot results and quantification of Drp1 and Drp1-p616. Each western blot was conducted for three times. **E**: Representative confocal images of immunofluorescence of Drp1 and mitochondria. Mitochondria were stained with mitotracker. Nuclei were stained with DAPI. Scale bar is 10 μm. ^∗∗^*P* < 0.01 vs. Ctrl; ^∗∗∗^*P* < 0.001 vs. Ctrl; **^##^***P* < 0.01 vs. HG.

Mitochondrial morphology is under elegant control of fission/fusion activities, and several proteins are responsible for it (Chan, 2012). So we measured the level of mitochondrial fusion proteins (Mfn1, Mfn2, and OPA1) and mitochondrial fission proteins (Drp1, MFF) by western blot. As is shown in **Figures 3A,B**, among proteins responsible for mitochondrial fusion, Mfn2 was downregulated obviously, while Mfn1 and OPA1 showed no statistic significant changes. Meanwhile, mitochondrial fission proteins, including Drp1 and MFF, were upregulated obviously (**Figures 3C,D**). These changes may account for mitochondrial over-fission. Besides, Drp1 is the most important protein for mitochondrial fission because it is the motor protein which provides energy to fission (Michalska et al., 2016). When Drp1 is phosphorylated at serine 616, its activation can induce more mitochondrial fission (Perdiz et al., 2017). We tested Drp1 phosphorylation and found that Drp1 phosphorylation level was upregulated significantly. Not surprisingly, berberine can attenuate imbalance of mitochondrial fission and fusion (**Figures 3A–D**) by inhibiting Drp1 phosphorylation. These results indicated that high glucose treatment can break up mitochondrial fission and fusion balance, and cause mitochondrial over-fission and fragmentation. Meanwhile, Drp1 is a cytoplasm protein. It can induce fission when translocating to mitochondria. So we did immunofluorescence to further investigate whether Drp1 translocated to mitochondria under high glucose treatment. As shown in **Figure 3E**, high glucose treatment made Drp1 translocate more to mitochondria, showing more co-localization with mitochondria, while berberine can prevent Drp1 translocate to mitochondria, indicating that berberine can not only decrease protein level of Drp1, but also keep Drp1 from mitochondria translocating, hence protect mitochondria from over-fission.

### Inhibiting Mitochondrial Fission Can Attenuate Hypertrophy

**FIGURE 4:**
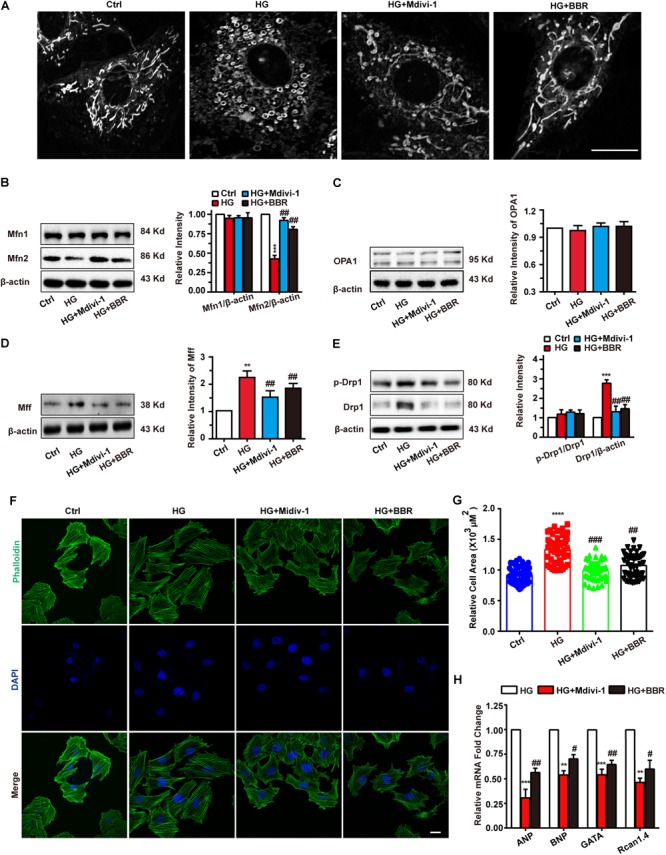
Berberine-inhibited mitochondrial fission can attenuate hypertrophy. **A**: Representative confocal images of mitochondria of H9C2 cells stained with mitotracker. Scale bar is 10 μm. **B**: Representative western-blot results and quantification of Mfn1 and Mfn2 **(B)**, OPA1 **(C)**, and MFF **(D)** when treated with Mdivi-1. Each western blot was conducted for three times. **E**: Representative western blot results and quantification of Drp1 and Drp1-p616. Each western blot was conducted for three times. **F**: Representative confocal images of H9C2 cells stained with FITC-labeled phalloidin to show its area. Scale bar is 10 μm. **G**: Quantification of H9C2 cell area in each group. For each group, *n* = 50–55 cells were quantified. **H**: Relative mRNA level of myocardium hypertrophy biomarkers quantified by real-time PCR. *n* = 3 for each group, each sample were measured in three replications. ^∗∗^*P* < 0.01 vs. Ctrl; ^∗∗∗^*P* < 0.001 vs. Ctrl; ^∗∗∗∗^*P* < 0.0001 vs. Ctrl; **^#^***P* < 0.05 vs. HG; **^##^***P* < 0.01 vs. HG;**^###^***P* < 0.001 vs. HG.

As is shown in **Figure 3**, berberine can inhibit mitochondrial over-fission. So we wondered whether the relief of hypertrophy by berberine is due to mitochondrial fission inhibition, so we used Mdivi-1, a selective Drp1 antagonist and can downregulate its fission activity, to investigate the relationship of mitochondrial fission and hypertrophy. As is shown in **Figure 4A**, Mdivi-1 prevented mitochondrial fragmentation under high glucose like berberine did. Western blot showed similar results (**Figures 4B–E**) as berberine, which displayed as decreased mitochondrial fission protein (Drp1 and MFF) and increased mitochondrial fusion (Mfn2). Mfn1 and OPA1 still showed no change under Mdivi-1 treatment, which suggested that Mfn1 and OPA1 may not be responsible for high glucose-induced mitochondria impairments.

Next, FITC-phalloidin was used to stain cytoskeleton. Interestingly, Mdivi-1 was indeed able to alleviate high glucose-induced H9C2 hypertrophy like berberine did (**Figures 4F,G**), and biomarkers of hypertrophy were downregulated by addition of Mdivi-1 (**Figure 4H**). We also tested mitochondrial functions like ATP production, MMP, and ROS production (**Supplementary Figure S2**) and got similar results like that of berberine. Hence, berberine attenuated H9C2 hypertrophy by inhibiting mitochondrial over-fission.

### High Glucose Treatment Impaired Mitogenesis Process and Downregulated Mitochondria Amount

**FIGURE 5:**
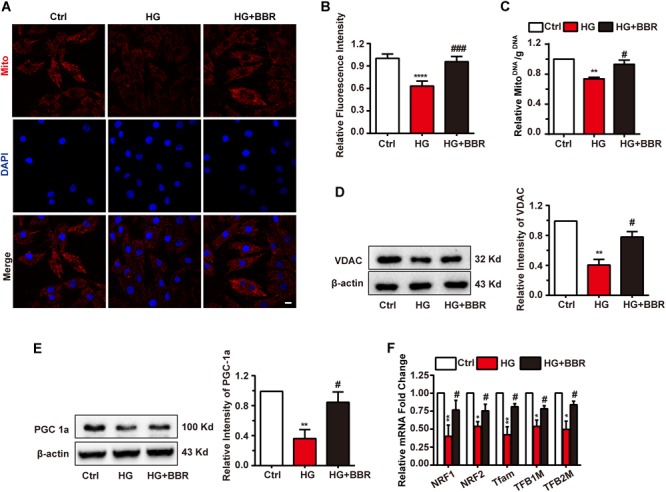
Berberine attenuated downregulate mitochondria amount and impaired mitogenesis process. **A**: Representative confocal image of mitochondria of H9C2 cells. Scale bar is 10 μm. **B**: Statistical analysis of relative mitotracker intensity. For each group, *n* = 60 cells were quantified. **C**: Relative mitochondrial DNA/g DNA ratio quantified by real-time PCR using COX1 as mitochondrial DNA loading control and 28SrRNA as g DNA loading control. *n* = 3 for each group, each sample were measured in three replications. **D**: Representative western blot results and quantification of VDAC. **E**: Representative western blot results and quantification of PGC1α. Each western blot was conducted for three times. **F**: Relative mRNA level of mitogenesis transcriptional factors quantified by real-time PCR. *n* = 3 for each group, each sample were measured in three replications. ^∗^*P* < 0.05 vs. Ctrl; ^∗∗^*P* < 0.01 vs. Ctrl; ^∗∗∗∗^*P* < 0.0001 vs. Ctrl; **^#^***P* < 0.05 vs. HG; **^###^***P* < 0.001 vs. HG.

Mitochondria are under tight and precise regulation during whole cell life. It was reported that cell can sense the state of mitochondria and did response to state changes (Ito and Ito, 2016). We found obvious decrease of mitochondria amount under the treatment of high glucose, and berberine was able to preserve mitochondria content (**Figures 5A,B**). To be confirmed, we measured mitochondria number by real-time PCR and western blot. The ratio of mitochondrial encoded DNA (COX1) and nuclear encoded gDNA (28SrRNA) can reflect the number of mitochondria (Lauritzen et al., 2015), and we found that high glucose treatment downregulated the ratio while berberine recovered it (**Figure 5C**). Then voltage dependent anion channel (VDAC) was used as mitochondrial internal control, western blot showed that VDAC decreased after high glucose treatment and berberine can reverse it (**Figure 5D**). Question was raised up with this phenomenon. As is known to all, mitochondria undergo ceaseless fission and fusion through the whole cell life, and its biogenesis is regulated by several factors, among which PGC1a (PPARG Coactivator 1a) is the most important one (Kalfalah et al., 2014). Hence we measured the level of PGC1a and found it is downregulated by high glucose treatment and recovered by berberine (**Figure 5E**). Then we did real-time PCR to investigate several transcript factors (TFs), including nuclear respiratory factor 1 (NRF1), nuclear respiratory factor 2 (NRF2), transcription factor A, mitochondrial (Tfam), mitochondrial transcription factor B1 (TFB1M), and mitochondrial transcription factor B2 (TFB2M), which are regulated by PGC1a and essential to mitochondrial biogenesis (Ploumi et al., 2017). As shown in **Figure 5F**, under the treatment of high glucose, these mitogenesis TFs were downregulated by high glucose, which may account for the decreased amount of mitochondria, and were reversed with the help of berberine. Hence high glucose treatment may not only have effects on mitochondrial fission and fusion process, but also decrease total mitochondria by impairing mitogenesis, while berberine can stimulate mitogenesis and help it recovering normal function.

### High Glucose Treatment Did Not Affect Initiation of Mitophagy, but Disturb Mitophagy Proceeding

**FIGURE 6:**
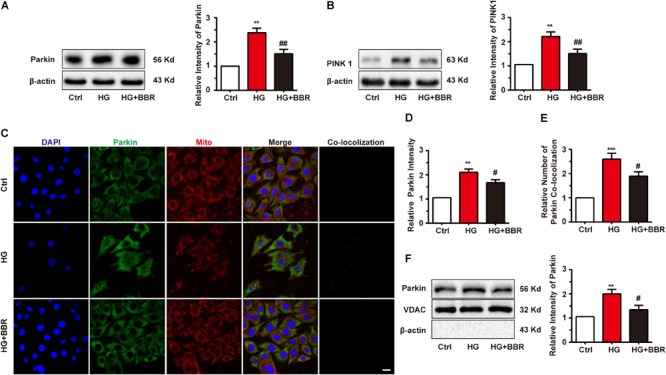
Berberine treatment recovered mitophagy proceeding. **A–B**: Representative western blot results and quantification of Parkin and PINK1. Each western blot was conducted for three times. **C**: Representative confocal images of immunofluorescence of Parkin and mitochondria. Parkin was labeled with primary antibody and was detected by FITC-labeled secondary antibody. Mitochondria were stained with mitotracker. Nuclei were stained with DAPI. Scale bar is 10 μm. **D**: Statistical analysis of Parkin puncta using ImageJ. For each group, 40–50 cells were measured. **E**: Co-localization analysis of Parkin and mitochondria using ImageJ. **F**: Representative western blot results and quantification of Parkin in purified mitochondrial. Each western blot was conducted for three times. ^∗∗^*P* < 0.01 vs. Ctrl; ^∗∗∗^*P* < 0.001 vs. Ctrl; **^#^***P* < 0.05 vs. HG; **^##^***P* < 0.01 vs. HG.

Mitogenesis and mitophagy are two sides of whole life of mitochondria. From above results we had found that mitogenesis was impaired by high glucose, which is one of the reasons that mitochondria decrease. We then asked whether mitophagy, the clearance process of mitochondria, was affected by high glucose. So we measured the level of Parkin and PINK1, the key initiation factor of mitophagy (Liu et al., 2014). Results were shown in **Figures 6A,B**. Parkin and PINK1 were obviously upregulated after high glucose treatment, indicating that the cell can sense damaged mitochondria and may initiate mitophagy. To confirm whether mitophagy was initiated by Parkin reorganization, we did immunofluorescence to test Parkin sub-cellular distribution. As to our speculation, Parkin translocated to mitochondria more under the treatment of high glucose, and berberine can inhibit Parkin translocation (**Figures 6C–E**). We further separate mitochondria of H9C2 cell and measured mitochondria-located PINK1 and Parkin, and found similar results as immunofluorescence (**Figure 6F**). This meant mitophagy initiation was not influenced by high glucose treatment.

**FIGURE 7:**
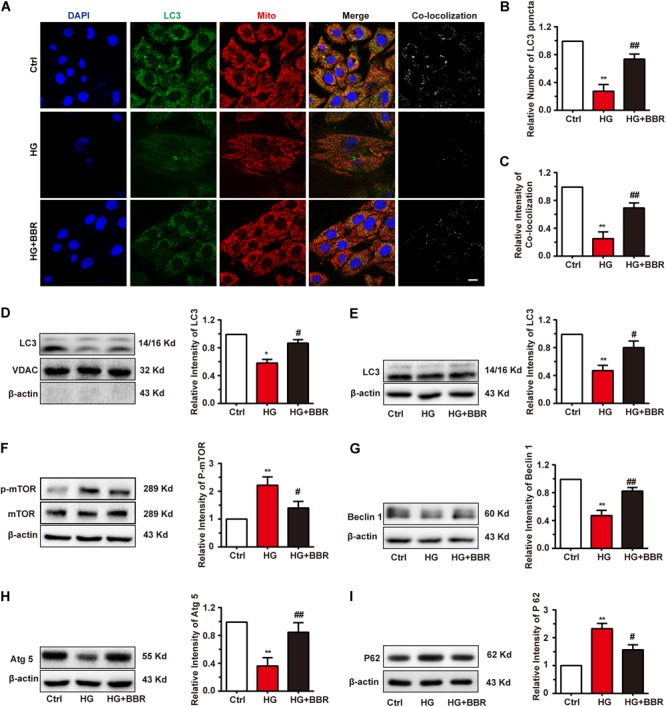
Berberine restored autophagic flux. **A**: Representative confocal images of immunofluorescence of LC3 and mitochondria. LC3 was labeled with primary antibody and was detected by FITC-labeled secondary antibody. Mitochondria were stained with mitotracker. Nuclei were stained with DAPI. Scale bar is 10 μm. **B**: Statistical analysis of LC3 puncta using ImageJ. For each group, 40–50 cells were measured. **C**: Co-localization analysis of LC3 and mitochondria using ImageJ. **D**: Representative western blot results and quantification of LC3 in mitochondria. Western blot results and quantification of LC3 **(E)**, mTOR **(F)**, Beclin1 **(G)**, Atg5 **(H)**, and P62 **(I)** in whole cell lysates. Each western blot was conducted for three times. ^∗^*P* < 0.05 vs. Ctrl; ^∗∗^*P* < 0.01 vs. Ctrl; **^#^***P* < 0.05 vs. HG; **^##^***P* < 0.01 vs. HG.

When Parkin translocate to mitochondria, it will induce the mitochondria degradation by the process called mitophagy (Liu et al., 2014). Finally, LC3-II-sequestered mitochondria, or the mitophagesome, will infuse with lysosome and degrade impaired mitochondria. Since the initiation of mitophagy was not disturbed by high glucose, we wondered whether the degradation of LC3-II-sequestered mitochondria would be affected by high glucose. Results of immunofluorescence revealed interesting results. The amount of LC3 was downregulated obviously (**Figure 7A**), and LC3-puncta number decreased significantly under the treatment of high glucose (**Figure 7B**), which indicated the decrease of formation of autophagysome. Besides, the co-localization analysis revealed that co-localization between mitochondria decreased significantly (**Figure 7C**). Western blot of separated mitochondria also had the similar results (**Figure 7D**). Berberine can upregulate the level of LC3 and LC3-puncta (**Figures 7A–D**), which indicated that it may alleviate the state of autophagy. These results suggested that although LC3 can still co-localize with mitochondria in a basic degree because low degree of LC3-II still can be detected, the total degree of mitophagy may still be downregulated due to decreased LC3 amount. But basic mitophagy level can still be detected; this may explain why the total amount of mitochondria still decreased.

### High Glucose Impaired Global Autophagy Level Due to Activation of mTOR

As is known, mTOR is the key modulating hub of cell autophagy state. When mTOR is inactivated, it will trigger autophagy or macrophagy. Since the total amount of LC3 and the dot number of LC3 decreased significantly by high glucose treatment (**Figures 7A,B**), we asked whether this is the results of downregulation of global autophagy level. As to our speculation, high glucose treatment activated mTOR by upregulating its phosphorylation level (**Figure 7F**). As a result, Beclin1 and Atg5 were downregulated (**Figures 7G,H**), both of which are key factors of autophagesome formation. Global level of LC3-II decreased as well (**Figure 7E**), which is consistent with the result of immunofluorescence, and indicated that sequestration and elongation of autophagesome were disturbed. Finally, the level of p62 increased (**Figure 7I**), as the result of inability of substrate degradation. Surprisingly, these changes were reversed by the treatment of berberine (**Figures 7E–I**). All of these results revealed that global autophagy level was impaired by high glucose, and berberine can activate autophagy and hence helped the degradation of detrimental substrates caused by high glucose, for example, impaired mitochondria.

### Berberine Targeted at AMPK to Protect H9C2 Cell From High Glucose-Induced Hypertrophy

**FIGURE 8:**
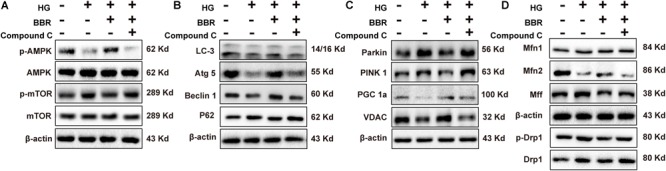
Berberine attenuated high glucose caused effects by activating AMPK. Representative western blot results of AMPK/mTOR signaling pathway **(A)**, autophagy-related protein **(B)**, mitophagy and mitogenesis-related protein **(C)**, mitochondrial dynamic-related protein **(D)** in compound C-treated H9C2 cell. Each western blot was conducted for three times.

It was wildly reported that berberine is an agonist of AMPK (Yerra et al., 2018). So we used compound C, a typical AMPK inhibitor, to investigate the anti-hypertrophy effect of berberine. As is shown in **Figure 8A**, AMPK was inactivated by high glucose, and berberine can activate AMPK, while this phenomenon can be canceled by compound C. This is consistent with previous reports that inactivation of AMPK will shut down autophagy (Li Y. et al., 2014). We then tested phosphorylation of mTOR and found that berberine-induced inhibition of mTOR was reversed by compound C (**Figure 8A**). We then checked other key autophagy factors mentioned above (Beclin1, Atg5, LC3, and p62), and found that compound C abrogated the effects berberine did (**Figure 8B**). These suggested that berberine upregulated autophagy by targeting AMPK.

It was reported that AMPK can also modulate PGC1a (Ning et al., 2018; Yu et al., 2018), so we also tested the level of PGC1a (**Figure 8C**) and found that compound C indeed canceled the upregulation of PGC1a caused by berberine. As a result, the level of VDAC decreased (**Figure 8C**), which showed that the amount of mitochondria decreased. Finally, we wondered whether the effects berberine did to mitochondrial fission and fusion proteins can also be abolished by compound C, so we measured their level (**Figure 8D**) and the results were consistent with our thought. Taken together, these results suggested that berberine protect mitochondria from high glucose treatment by targeting at AMPK.

## Discussion

Diabetic cardiovascular injury is a serious complication in diabetes. The emergence of cardiac implications in many diabetes patients is because of poor control of hyperglycemia. It is confirmed that hyperglycemia is usually accompanied with the occurrence of myocardium hypertrophy (Hu et al., 2017; Toedebusch et al., 2018). Diabetes-induced myocardium hypertrophy has affirmed by most scientists that research in cardiovascular field (Adeghate and Singh, 2014), but therein the molecular mechanism is not well understood. Progressive impairment of the heart is its main manifestation in diabetes-induced myocardium hypertrophy, which shows an increase of myocardial cell volume and upregulation of hypertrophy gene. Slowing down the process of myocardium hypertrophy and reducing the damage caused by myocardium hypertrophy have become a goal that scientists are trying to find in diabetes.

Berberine, a monomeric compound derived from traditional Chinese medicine *Coptis chinensis*, has shown hypoglycemic effect *in vitro* and *in vivo* (Chang et al., 2015; Chen et al., 2017). It has also been shown that berberine can relieve the occurrence of high glucose-induced insulin resistance in a safe range (Shen et al., 2012; Geng et al., 2016). The present study investigated the effects of berberine on high glucose-induced myocardium hypertrophy. Furthermore, we showed that berberine ameliorated high glucose-induced cardiomyocyte injury via AMPK signaling activation to stimulate mitochondrial biogenesis and restore autophagy flux in H9C2 cells.

To be obviously, the hypertrophy genes and cell area were upregulated when H9C2 myocardial cells were exposed to 50 mM high glucose environment (**Figure 1**), while with the help of berberine this phenomenon was reversed (**Figure 1**). The occurrence of myocardium hypertrophy is closely related to mitochondrial fission, which has been reported in several literatures (Pennanen et al., 2014; Tang et al., 2014). This occurrence was also appeared in our research when H9C2 cells were incubated for 24 h under 50 mM high glucose condition. However, after the administration of berberine, not only the damaged mitochondrion morphology has been improved, but also the mitochondrial function has been well restored (**Figure 2**). To clarify the mechanisms, we detected the level of proteins responsible for mitochondrial fusion and fission. As shown in **Figure 3**, berberine antagonized the decrease in expression of Mfn2, the outer membrane fusion proteins. Meanwhile, the expression of another outer membrane fusion protein, Mfn1, and the inner membrane fusion protein, OPA1, showed no change among control group, berberine-treated group, and high glucose-treated H9C2 cell group. This indicates that high glucose-induced mitochondrial damage is mostly related to its outer membrane fusion dysfunction, rather than interrupting inner membrane fusion process; and high glucose treatment mainly did detrimental effects to mitochondrial outer membrane fusion through Mfn2. Besides, we found the two proteins, MFF and Drp1, which are related to the fission of mitochondrial, were all significantly upregulated in high glucose-treated H9C2 cells, yet not in berberine treatment (**Figures 3C–E**). Moreover, treating H9C2 with Midiv-1, the Drp1 inhibitor, ameliorated the mitochondrial functions and morphology in high glucose-treated cells. The similar phenomenon was also observed in the berberine treatment group (**Figures 4A–E**). These results indicated that the mitochondrion function and morphology were improved by berberine via inhibiting mitochondrial fission and boosting the mitochondrial fusion. Additionally, we also found that inhibition of mitochondrial fission was negative correlation with the occurrence of myocardium hypertrophy in glucose-treated H9C2 cells (**Figures 4F–H**). It was reported that mitochondrial dynamics have important effects to cardiovascular diseases (Duncan, 2011). Duncan (2011) reported that inhibiting mitochondrial fission can improve the prognosis of diabetes myocardial infarction mice, and here we showed that hypertrophy is also related to mitochondrial dynamics. Hence manipulating mitochondrial dynamics may be a promising way to treat cardiovascular events.

The energy for mitochondrial fission process is mainly provided by the GTPase Drp1. Drp1 was translocated to mitochondria after high glucose treatment, and we further found that Drp1 activity was also upregulated by phosphorylation at serine 616. It was reported that GSK3β can phosphorylate Drp1 at serine 616 (Yan et al., 2015), so we measured GSK3β activity and found that its phosphorylation at serine 9, the inhibitory phosphorylation site, was downregulated, and GSK3β showed more translocation to mitochondria by high glucose treatment (**Supplementary Figure S3**). So GSK3β is a modulatory enzyme that regulates mitochondrial fission through Drp1 activity. Besides, Liang et al. (2018) has reported that GSK3β activity is negatively regulated by AMPK. Berberine can activate AMPK, which can phosphorylate GSK3β at serine 9 to inhibit its activation, this gives new insight to the mechanism that berberine can alleviate mitochondrial fission and hence hypertrophy.

Mitochondrial fragmentation is intimately correlated to mitogenesis. Mitogenesis is a crucial process in cells for providing new mitochondria to satisfied energy metabolism acquirement (Kalfalah et al., 2014). It was reported that PGC1a is a vital co-transcription factor responsible for mitogenesis (Ploumi et al., 2017). It can cooperate with several transcription factors like NRF1, NRF2, and modulate their transcription activities, so as to activate mitogenesis. In HG-treated H9C2 cells, down regulated PGC1a was accompanied by decrease in mitochondria amount and mitogenesis transcription factors mRNA level, namely that mitogenesis was strongly impaired. Fortunately, berberine can stimulate mitogenesis by increasing PGC1a and restoring the level of key mitogenesis transcription factors mRNA level, hence generating new mitochondria to adapt to high glucose environment.

Like two sides of one coin, mitochondrial fragmentation can not only stimulate mitogenesis, but can also trigger mitophagy, or the process of mitochondria clearance. The most distinguished pattern of mitophagy is through PINK1/Parkin pathway. As was reported, Parkin can recognize dysfunctional mitochondria by PINK1 recruitment, and dysfunctional mitochondria will be marked with ubiquitin and recognized by LC3, finally sent to lysosome for degradation (Liu et al., 2014). Our results showed corresponding results that PINK1/Parkin was indeed upregulated and more Parkin translocate to mitochondria under high glucose environment, which indicate the initiation of mitophagy. However, very to our surprise, when we detect the level of LC3, which represent the degradation level of mitochondria, it was downregulated remarkably, and this is inconsistent to our original speculation that the decreased amount of mitochondria is due to inefficient mitogenesis and excessive mitophagy. But after deeper investigation, we found that LC3-II was decreased but not vanished, co-localization of LC3 and mitochondria was unchanged, and these data showed that mitophagy still carry on at basic level under high glucose environment. This gives reasonable explanation to the decrease of mitochondria amount.

Besides, obvious decrease of LC3 sent us to think about the global autophagy flux state under high glucose treatment. Not unexpectedly, AMPK was inactivated by high glucose, which is consistent with previous reports (Zhu et al., 2017; Liang et al., 2018). As a result, mTOR activity was upregulated, which set barrier to the initiation of autophagy, hence decreased the total LC3-II level. Berberine can directly activate AMPK and restore the level of Beclin1, Atg5, and LC3. As a result, the disturbance of autophagy flux by high glucose can be alleviated by berberine. What’s more, the restoration of LC3 level can help Parkin-labeled dysfunctional mitochondria to lysosome degradation, otherwise these dysfunctional mitochondria will do harm to cell through leakage of cytochrome C and trigger of apoptosis.

It is widely reported that AMPK is inactivated under HG or diabetes. This is an interesting phenomenon worth investigation. As is known that AMPK is the sensor of cell energy state. AMPK can be regulated by several upstream regulators like LKB1 (Woods et al., 2003), CaMKKII, and PP2A (Towler and Hardie, 2007). It is considered as a protective strategy that AMPK is inactivated under the stress of high glucose environment, because AMPK can stimulate GLUT4 translocating from cytoplasm to cell membrane, hence transporting more glucose into the cytoplasm. GLUT4 is the most abundant form of glucose transporters in cardiomyocyte, and GLUT4 deletion can induce myocardium hypertrophy *in vivo* (Abel et al., 1999). We also have found that GLUT4 was downregulated in mRNA level and this be reversed by berberine (data not shown). This can be another possible mechanism behind the anti-hypertrophy effect berberine does.

**FIGURE 9:**
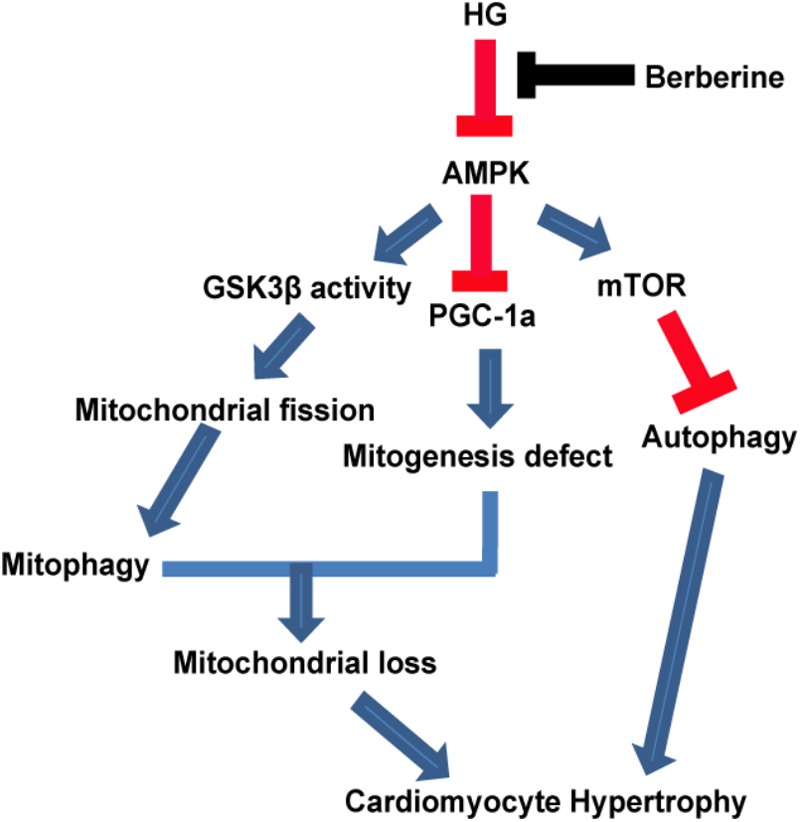
A functional model of berberine protecting effects against high glucose-induced myocardium hypertrophy.

Therefore, our study provided evidence that berberine could prevent hypertrophy via activating AMPK to stimulate mitochondrial biogenesis and restore autophagy flux disturbance, and the working model is shown in **Figure 9**.

## Author Contributions

WH drafted the manuscript, and carried out cell culture and biochemical measurements. BH and JC performed western blotting. LX, XW, and QZ carried out cell culture and treatments. BW, TL, and YW participated in the preparation of the manuscript. QC participated in the design and preparation of the manuscript. JC conceived the study and participated in the coordination and preparation of the manuscript. All authors read and approved the final draft.

## Conflict of Interest Statement

The authors declare that the research was conducted in the absence of any commercial or financial relationships that could be construed as a potential conflict of interest.
