# Supplementary material for: Berberine Ameliorates High Glucose-Induced Cardiomyocyte Injury via AMPK Signaling Activation to Stimulate Mitochondrial Biogenesis and Restore Autophagic Flux
Source: Front Pharmacol. 2018 Oct 3;9:1121. doi: 10.3389/fphar.2018.01121 (PMC6178920; doi:10.3389/fphar.2018.01121)
Supplement: Supplementary file 1 [file Table_1.DOCX]

Berberine ameliorates high glucose-induced cardiomyocyte injury via AMPK signaling activation to stimulate mitochondrial

biogenesis and restore autophagic flux

**Running title:** Berberine ameliorates cardiomyocyte hypertrophy

Weijian Hang^1,5^, Benhong He^4^, Jiehui Chen^1^, Liangtao Xia^1^, Bing Wen^1^, Tao Liang^1^, Xu Wang^1^, Qianying Zhang^1^, Yue Wu^1^, Qingjie Chen^1,6*^, Juan Chen^1,2,3*^

^1^ Department of Biochemistry and Molecular Biology, School of Basic Medicine and the Collaborative Innovation Center for Brain Science, Tongji Medical College, Huazhong University of Science and Technology, Wuhan, 430030, Hubei, China.

^2^ Institute for Brain Research, Huazhong University of Science and Technology, Wuhan, 430030, Hubei, China

^3^ Key Laboratory of Neurological Disease of National Education Ministry, Tongji Medical College, Huazhong University of Science and Technology, Wuhan, 430030, Hubei, China

^4^ Department of Cardiovascular Medicine, Lichuan People's Hospital, Lichuan, 445400, Hubei, China.

^5^ Hubei Key Laboratory of Genetics and Molecular Mechanisms of Cardiological Disorders, Huazhong University of Science and Technology

^6^ New products of TCM Senile Diseases Co-Innovation Center of Hubei, Basic Medical Sciences College, Hubei University of Chinese Medicine, Wuhan 430065, Hubei, China

**^*^Corresponding authors:**

Email addresses: [chenjuanlinda69@163.com](mailto:chenjuanlinda69@163.com) (J. Chen); [chenqingjie8858@163.com](mailto:chenqingjie8858@163.com)

**Key word:** berberine, cardiomyocyte hypertrophy, mitochondrial, high glucose, fragmentation, diabetes mellitus

## Supplementary methods

## CCK8 cell viability assay

CCK8 cell viability assay carried according to the manufacturer’s protocol (Beyotime, #C0039). Briefly, about 1000 cells were planted into one well of 96-well plate. After the cells were attached, cells were refreshed with 200ul medium with different concentration of glucose or berberine for 24h. Then 20ul CCK8 working solution were added into each well and the plate were incubated in 37°C for 30min. Then the optical density was measured with microplate reader (Thermo) at 450nm.

## LDH quantification assay

The LDH level of medium were measured according to the manufacturer’s protocol (Beyotime, #C0016). Briefly, 120ul of medium were mixed with 60ul LDH assay working solution and incubated at room temperature for 15min avoiding from light. Then the optical density was measured with microplate reader (Thermo) at 490nm.

Fig.S1


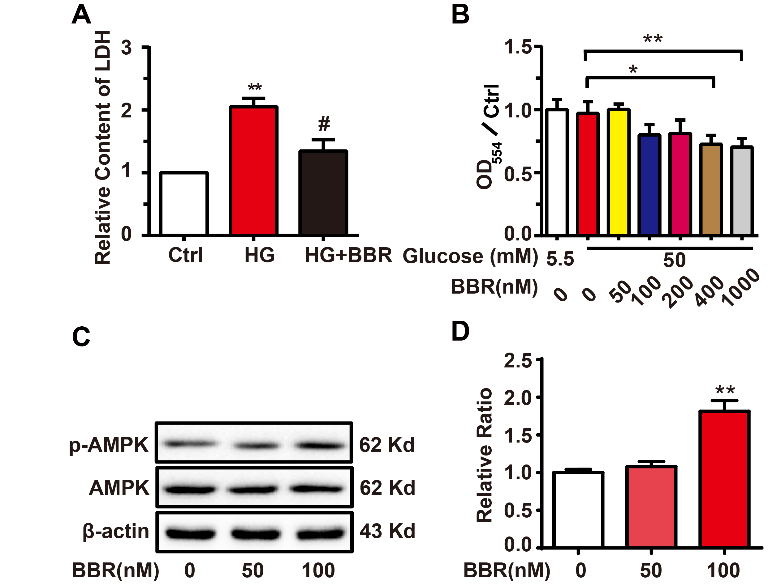


Fig.S1 Berberine is safe to H9C2 cells under 100nM concentration and is sufficient to activate AMPK. (A). Medium LDH level of HG and 100nM berberine. Each group were set 6 duplications, and the experiment were carried for 3 times. (B). CCK8 cell viability assay of different concentrations of berberine. Each group were set 6 duplications, and the experiment were carried for 3 times. (C-D). Representative Western-blot results and quantification of AMPK and p-AMPK Thr172. Each western blot was conducted for 3 times. ^*^*P* <0.05 vs. Ctrl; ^**^*P* <0.01 vs. Ctrl; **^#^***P* <0.05 vs. HG.

Fig.S2


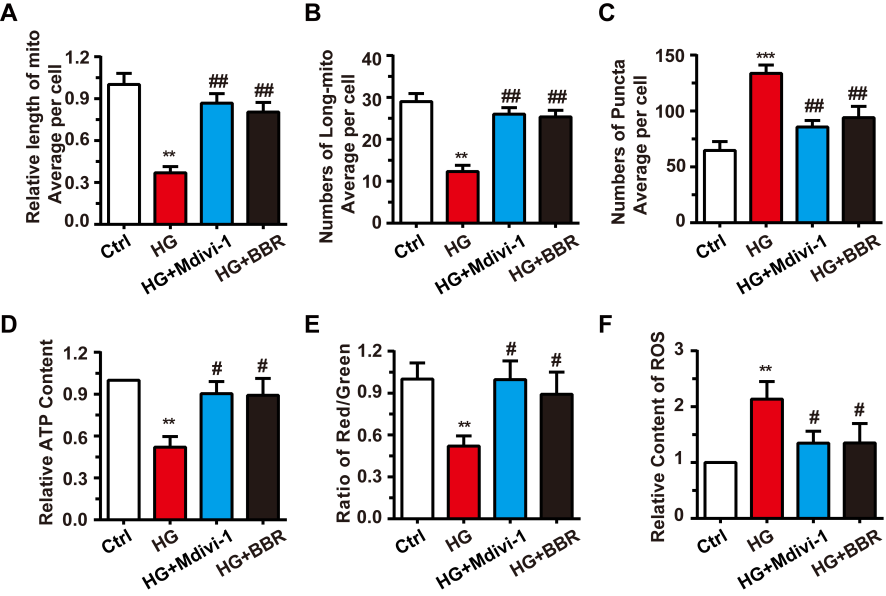


Fig.S2 Inhibiting mitochondria fission can attenuate high glucose induced mitochondrial dysfunction. Statistical analysis of mitochondria length (A), mitochondria area (B) and mitochondria puncta (C) using Image J, for each group, n=40-50 cells were analyzed. D: Relative ATP production was measured. E: Relative mitochondrial membrane potential (MMP) were measured by JC-1 and were quantified using the ratio of red and green fluorescence intensity. F: Relative ROS level were measured using DCFH-DA and were quantified using green fluorescence intensity. for each group, >10 fields were measured. ^**^*P* <0.01 vs. Ctrl; ^***^*P* <0.001 vs. Ctrl; **^#^***P* <0.05 vs. HG; **^##^***P* <0.01 vs. HG.

Fig.S3


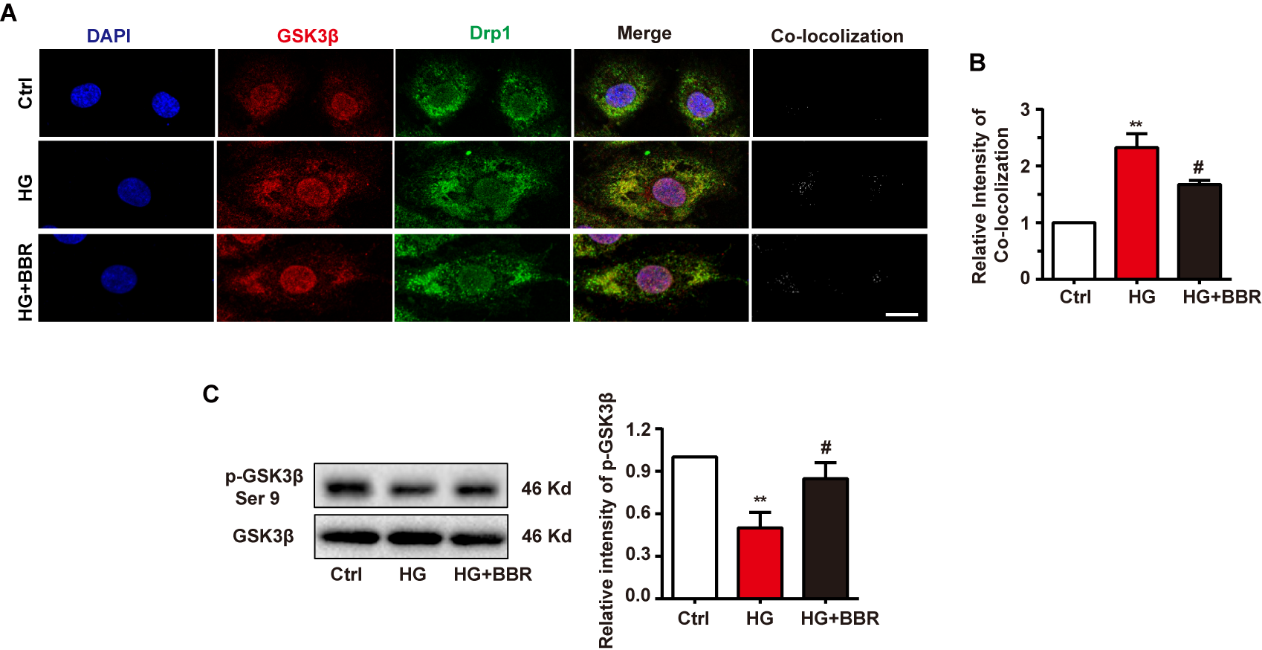


Fig.S3 A: Representative confocal images of immunofluorescence of GSK3β and Drp1. GSK3β was labelled with mouse primary antibody and was detected by Cy3-labelled secondary goat anti-mouse antibody, while Drp1 was labelled with rabbit primary antibody and was detected by FITC-labelled secondary goat anti-rabbit antibody. Nuclei were stained with DAPI. Scale bar is 10um. B: Co-localization analysis of GSK3β and Drp1 using Image J. C. Representative Western-blot results and quantification of GSK3β and p-GSK3β Ser9. Each western blot was conducted for 3 times. ^**^*P* <0.01 vs. Ctrl; **^#^***P* <0.05 vs. HG.
