# Supplementary figures and images for: Berberine Ameliorates High Glucose-Induced Cardiomyocyte Injury via AMPK Signaling Activation to Stimulate Mitochondrial Biogenesis and Restore Autophagic Flux
Source: Front Pharmacol. 2018 Oct 3;9:1121. doi: 10.3389/fphar.2018.01121 (PMC6178920; doi:10.3389/fphar.2018.01121)

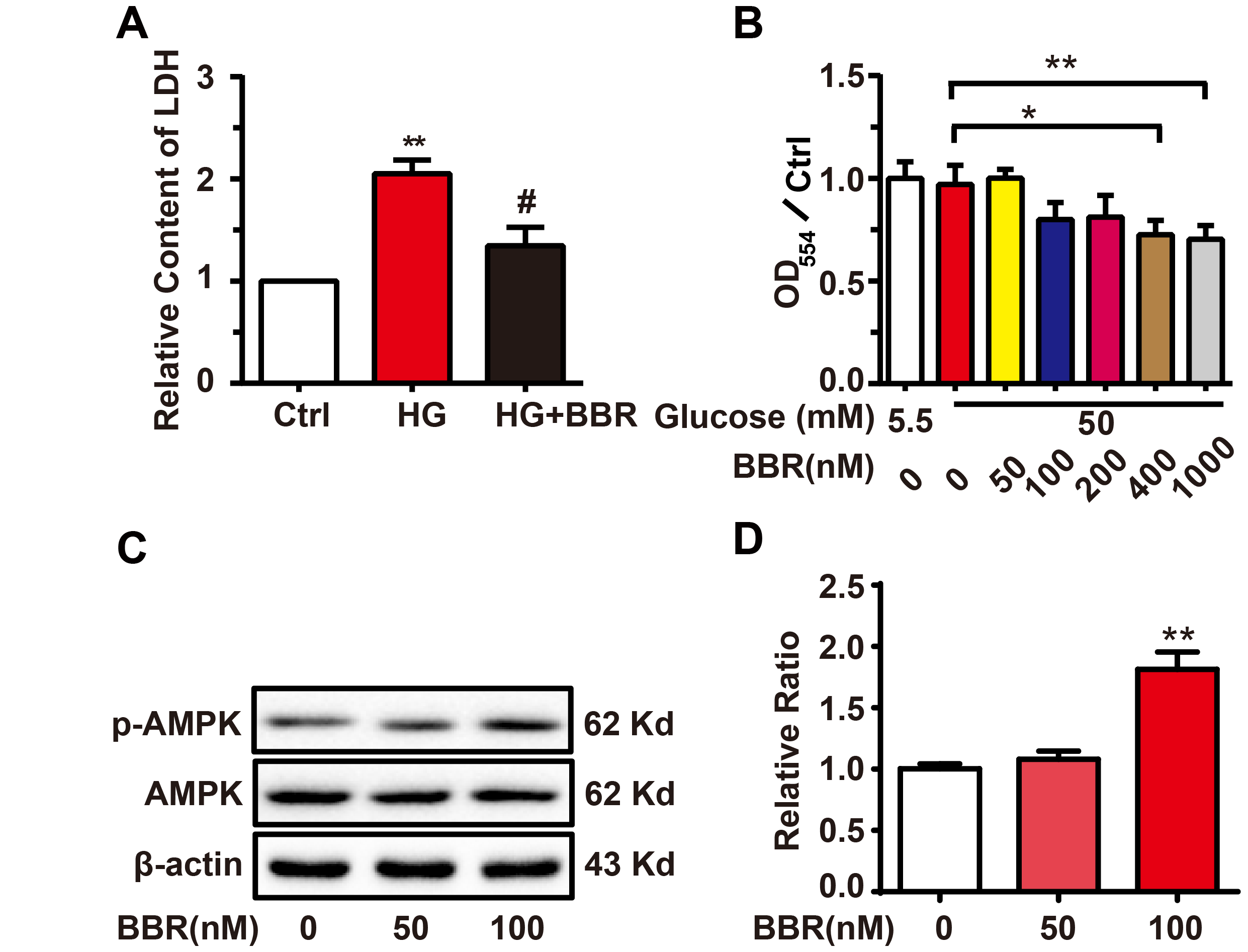

Supplement: Supplementary file 2 [file Image_1.TIF]

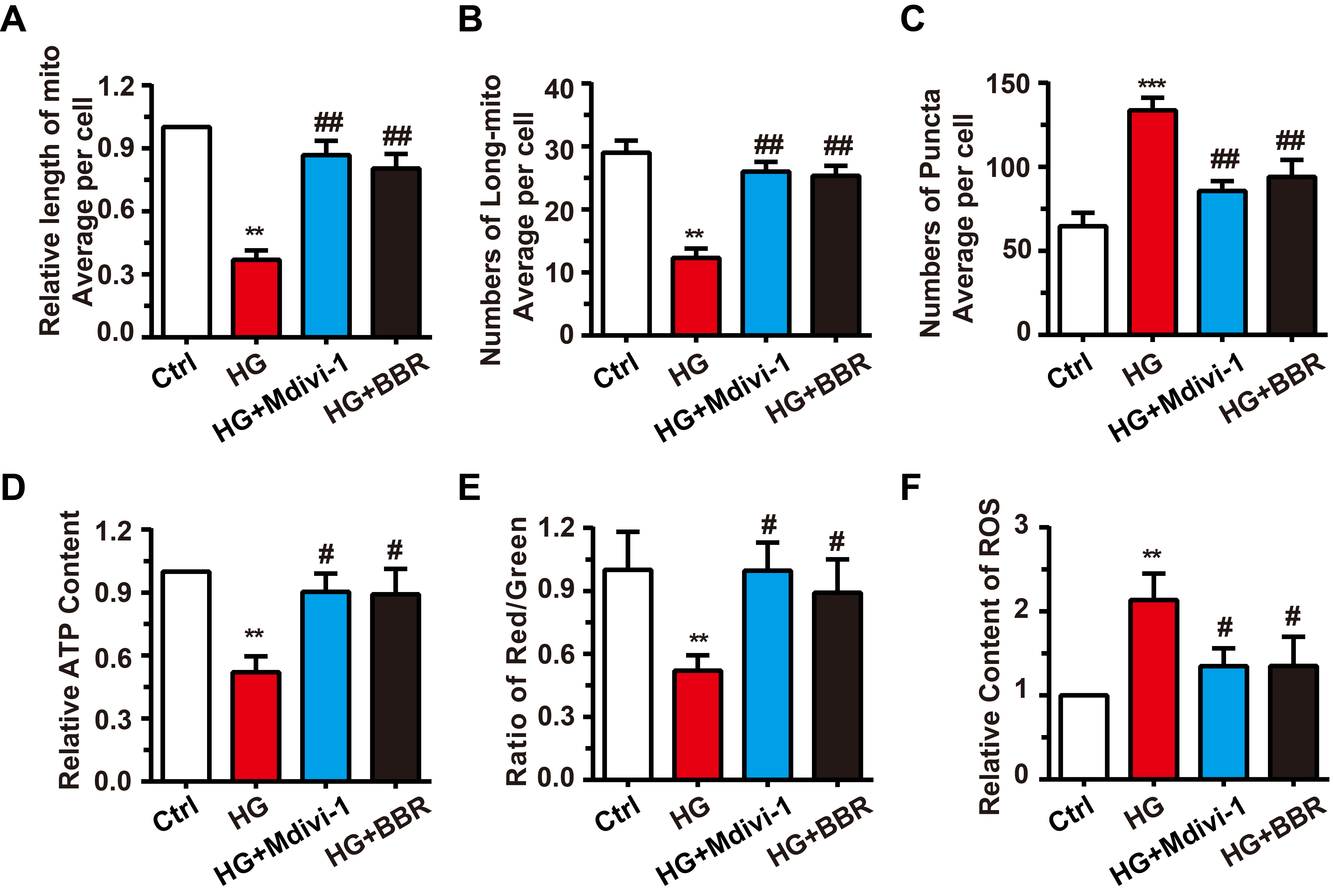

Supplement: Supplementary file 3 [file Image_2.TIF]

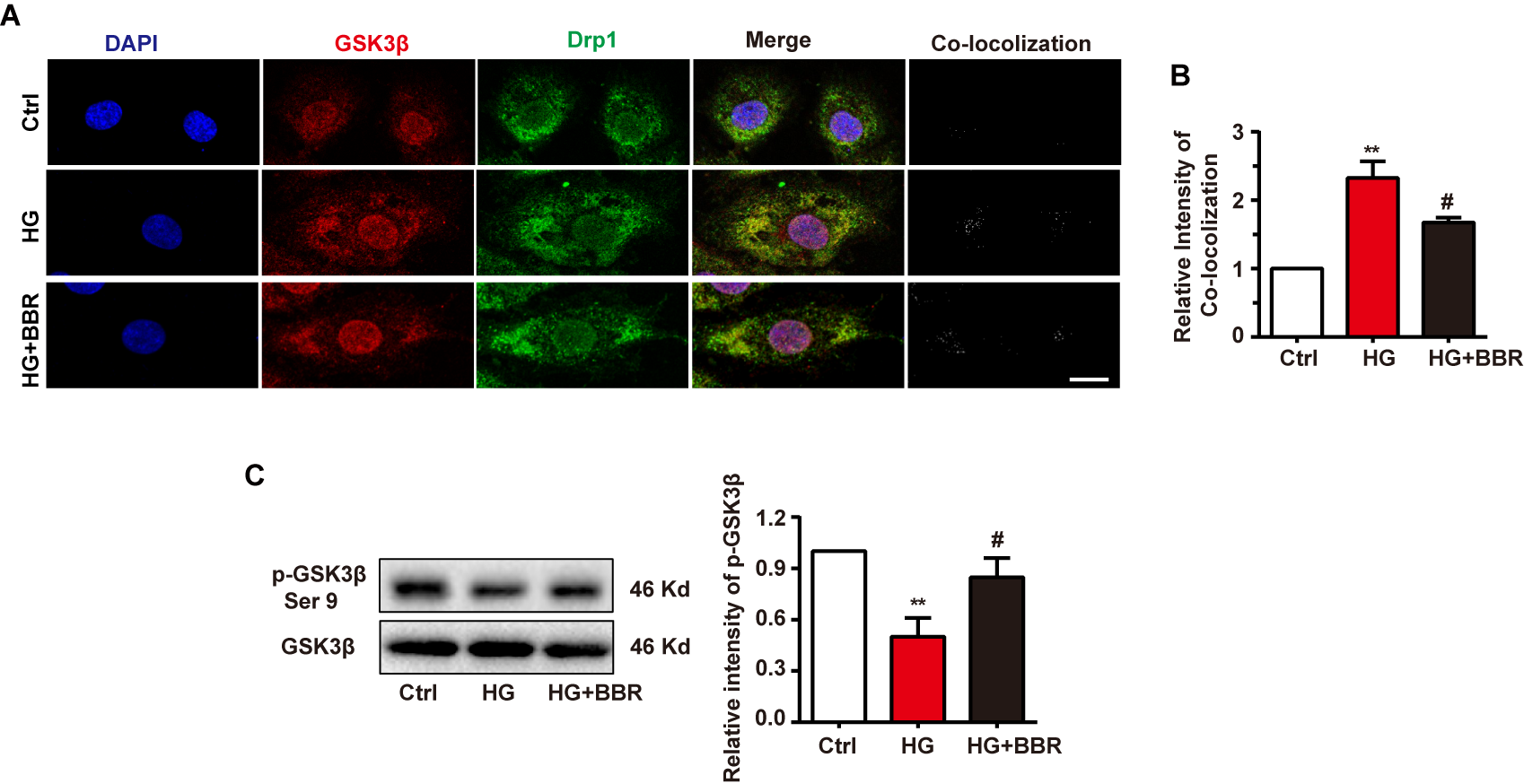

Supplement: Supplementary file 4 [file Image_3.TIF]
